# Quantifying Microsatellite Mutation Rates from Intestinal Stem Cell Dynamics in Msh2-Deficient Murine Epithelium

**DOI:** 10.1534/genetics.119.302268

**Published:** 2019-05-24

**Authors:** Joseph Christopher, Ann-Sofie Thorsen, Sam Abujudeh, Filipe C. Lourenço, Richard Kemp, Paul K. Potter, Edward Morrissey, Lee Hazelwood, Douglas J. Winton

**Affiliations:** * Li Ka Shing Centre, Cancer Research UK Cambridge Institute, University of Cambridge, CB2 0RE, United Kingdom; † Department Biological and Medical Sciences, Faculty of Health and Life Sciences, Oxford Brookes University, OX3 0BP, United Kingdom; ‡ MRC Weatherall Institute of Molecular Medicine, University of Oxford, OX3 9DS, United Kingdom

**Keywords:** microsatellite, mouse, intestine, mutation, Msh2

## Abstract

Microsatellite sequences have an enhanced susceptibility to mutation, and can act as sentinels indicating elevated mutation rates and increased risk of cancer. The probability of mutant fixation within the intestinal epithelium is dictated by a combination of stem cell dynamics and mutation rate. Here, we exploit this relationship to infer microsatellite mutation rates. First a sensitive, multiplexed, and quantitative method for detecting somatic changes in microsatellite length was developed that allowed the parallel detection of mutant [CA]_n_ sequences from hundreds of low-input tissue samples at up to 14 loci. The method was applied to colonic crypts in *Mus musculus*, and enabled detection of mutant subclones down to 20% of the cellularity of the crypt (∼50 of 250 cells). By quantifying age-related increases in clone frequencies for multiple loci, microsatellite mutation rates in wild-type and Msh2-deficient epithelium were established. An average 388-fold increase in mutation per mitosis rate was observed in Msh2-deficient epithelium (2.4 × 10^−2^) compared to wild-type epithelium (6.2 × 10^−5^).

SOMATIC mutational burden is a key driver in the development of cancer. Recent studies have revealed the extent to which acquired burden results in genetic mosaicism within the colonic epithelium of individuals due to the combined effects of mutation, stem cell dynamics, and selection (Tomasetti *et al.* 2013; Nicholson *et al.* 2018). However, the extent to which individuals differ in mutational burden, and, therefore, their risk of developing cancer remains largely unknown. One exception is Lynch syndrome patients in which germline mutation of genes regulating DNA mismatch repair (MMR) predisposes affected individuals to a >80% lifetime risk of developing colorectal cancer (CRC).

Lynch syndrome shows an autosomal-dominant pattern of inheritance and is caused by mutations in multiple different genes, including *Msh2*, mutation of which leads to the most severe phenotype (Jiricny 2006; Strafford 2012). Defective MMR causes hypermutation commonly recognized by microsatellite instability (MSI), and is observed in 12–17% of sporadic CRCs (Yuza *et al.* 2017). MSI^+^ CRCs are more responsive to immunotherapies (Le *et al.* 2015; Yuza *et al.* 2017), display restricted growth characteristics (Germano *et al.* 2017), and have had potentially therapeutic genetic susceptibilities identified (Behan *et al.* 2019; Chan *et al.* 2019; Lieb *et al.* 2019). A more favorable prognosis associated with MMR-deficiency is likely due to hypermutation increasing the frequency of neo-antigens and the recognition of cancer cells by the immune system (Gryfe *et al.* 2000; Goyal *et al.* 2016; Rizvi *et al.* 2016). Consequently, identifying MSI loci that correlate with the hypermutator phenotype is of clear therapeutic importance (Cortes-Ciriano *et al.* 2017).

There is a lack of methods for quantifying microsatellite mutation rates in somatic tissues. This is, in part, due to difficulty in classifying microsatellite length within mixed populations restricting analyses to detecting the presence or absence of any given allele rather than quantifying their relative proportions (Carlson *et al.* 2015; Cortes-Ciriano *et al.* 2017). The technical challenge in sequencing microsatellites, particularly from low input amounts of DNA template, is polymerase error due to “stuttering” that leads to mixed lengths of microsatellite during amplification and sequencing. This error appears to be greater with less complex (mononucleotide or dinucleotide) tracts or longer microsatellites, likely reflecting the inherent mutability of these loci *in vivo* (Sia *et al.* 1997; Baptiste *et al.* 2013; Lang *et al.* 2013). Consequently, many studies focus on shorter microsatellites, or those containing tetranucleotide repeats that, despite having lower mutation rates, are commonly used for human genotyping studies (Cortes-Ciriano *et al.* 2017).

Estimates of microsatellite mutation rate varies widely depending on tract length and nucleotide composition (Baptiste *et al.* 2013). Through *in vitro* and *in vivo* estimation, the mutation rate in [CA]_n_ microsatellites has been shown to be 3.7 × 10^−6^, 2.4 × 10^−5^, and 1.6 × 10^−4^ mutants per cell per generation in [CA]_8_, [CA]_17_ and [CA]_30_ respectively in an *in vitro* mouse model (Yamada *et al.* 2002), and 1.1 × 10^−4^ mutants per mitosis in a transgenic mouse [CA]_30_ microsatellite (Kozar *et al.* 2013). These estimates show that the rate of microsatellite change is directly linked to the number of repeat units.

The crypts of the colonic epithelium are glandular structures containing a multiplicity of stem cells at their base that give rise via proliferative progeny to the different differentiated intestinal cell types (Vermeulen and Snippert 2014). Within each crypt, stem cells are replaced by a stochastic process that is characterized by recurrent extinctions and expansions as stem cell compete with their neighbors at the point of cell division. This was recognized by analysis of the changing size distribution of stem cell derived clones over time that ultimately results in monoclonal conversion of the crypt such that it is populated by the progeny of a single victorious stem cell (Lopez-Garcia *et al.* 2010; Snippert *et al.* 2010). Where clones can be visualized, this process is recognized by transient oligoclonal crypts and their time- or age-related resolution to monoclonality. In the murine colonic epithelium, the parameters that define the clonal dynamics leading to monoclonal conversion have been inferred, and depend on two variables: the number of functional stem cells and the rate of their replacement.

Previously, we have described a transgenic mouse line in which reporter expression is obtained only after a change in repeat tract length (Kozar *et al.* 2013). In these *Rosa26-[CA]_30_-eYFP* animals, yellow fluorescent protein (YFP) expression requires spontaneous mutation in a hemizygous microsatellite. When mutation leads to YFP expression, the subsequent fate of the expressing cell and the clone that it supports is dictated by the outcome of stem cell competition described above. Only YFP^+^ clones that successfully drift to monoclonality within a crypt, recognized as wholly populated crypts (WPCs), become permanently fixed within the epithelium. YFP^+^ clones in transition are recognized as crypts that are only partly YFP^+^ (PPCs). The frequency of PPCs and WPCs relies on functional stem cell number, stem cell replacement rate, and the mutation rate at the observed locus. A consequence of the stem cell organization within the crypt is that clone dynamics act to amplify somatic variants at the cellular level. This makes high-throughput sequencing of single clonal units feasible. Here, we identify that knowledge of intestinal stem cell dynamics and accurate detection of age-related changes in native microsatellite lengths by targeted sequencing offers a novel route to infer *in vivo* mutation per mitosis rates in a locus-specific manner.

## Materials and Methods

### Mice

Mice were of the C57BL/6 strain. The *Rosa26-[CA]_30_-eYFP* (Kozar *et al.* 2013) and *Villin-CreER2* (El Marjou *et al.* 2004) mice have been described previously and were maintained in a hemizygous state on a C57BL/6 background. The *Msh2^fl/fl^* were obtained from Paul Potter, MGI:101816 (Friedel *et al.* 2007). All mice studied were cared for in the Cancer Research UK Cambridge Institute Biological Resource Unit (CRUK-CI BRU) according to UK Home Office guidelines. Genotyping was outsourced to Transnetyx (Cordova, TN).

### Crypt isolation

Colonic crypts were isolated from murine tissues based on a previously described method based on EDTA chelation and physical agitation, which releases crypts in a series of timed fractionations that are assessed under a dissecting microscope (Bjerknes and Cheng 1981). Fractions enriched in single crypts were pooled.

The single crypt enriched suspension was pelleted at 1200 rpm for 2 min. Once pelleted, media was removed and the crypts were resuspended in 50 ml cold PBS. Under a low power dissecting microscope, 400 μl of enriched crypt suspensions were spread on a siliconized (Sigmacote, SL2-25ML; Sigma-Aldrich) glass plate. Single crypts were then drawn into a micropipette using a mouth siphon before being moved through four PBS droplets for washing and visual identification of any cellular contaminants. Finally, the crypt was expelled into 6 μl of alkaline lysis buffer in a lobind eight-well PCR strip.

Crypts were then lysed via the following temperature cycling protocol: 50° 3 hr, 75° 20 min, 80° 5 min, 4° hold. The alkaline lysis buffer was neutralized using 6 μl neutralizing buffer. Each crypt lysate was divided into halves and processed separately.

Tail DNA was sampled and eight replicates processed in parallel to produce reference distributions for each mouse.

### Microsatellite plasmids

Two native loci were amplified from mouse genomic DNA isolated from tail samples using the Q5 polymerase (NEB) and primers incorporating restriction sites. The samples were amplified using the following protocol: 98° for 30 sec, then 60 cycles of 98° for 10 sec, 59° for 20 sec then 72° for 20 sec before a final extension of 72° for 2 min. The PCR product was ligated into the pUC19 plasmid (GenBank: M77789; Addgene) following *Bam*HI and *Hin*dIII digestions. Following transformation into XL-1 Blue Competent Cells (200249; Agilent) and selection, purified plasmids isolated from expanded colonies were screened by Sanger sequencing (Source BioScience, Nottingham, UK) using M13 primers. The number of [CA] repeats was counted manually.

### Primer design

A custom Perl script was developed to identify the location of all [CA]_30_ microsatellites within the mm9 genome. The output of this script also created a plain text file that was formatted for input into the online primer design tool BatchPrimer3 (You *et al.* 2008), with input of a 70 bp sequence flanking either side of the [CA]_30_.

The default BatchPrimer3 variables were used for all primer design, except for stipulating that the forward and reverse primers must be between 20 and 70 bp from their respective end of the [CA]_30_ tract. As a result, the maximum amplicon length was 200 bp and the minimum amplicon length was 100 bp with the optimal amplicon length stipulated as 180 bp. Each predicted amplicon was manually curated to screen out any locus with highly repetitive flanks that would have reduced the likelihood of ontarget amplification. Primers were tested in singleplex for PCR product generation at the expected length assessed by gel electrophoresis. The online tool MultiPLX 2.1 (Kaplinski and Remm 2015) was used to determine optimal multiplex PCR groups from primers successfully tested in singleplex. The final set of loci used for analysis of wild-type and Msh2-deficient crypts are summarized in Supplemental Material, Table S2.

Initially, the CS1/CS2 NGS adaptor system was added to the end of all primer pairs to allow compatibility with the Fluidigm barcoding system allowing pooling of up to 384 sequencing libraries. This system was used for all singleplex amplification and sequencing. For multiplexing, an inhouse designed NGS adaptor system, named “M13” adaptors, was used. These adaptors are compatible with an inhouse designed barcoding system allowing the pooling of up to 384 sequencing libraries while reducing primer-dimer contamination from multiplexed PCR.

### Library preparation

Loci containing microsatellites were amplified in the following reaction mixture: NEB High Fidelity buffer diluted to 1× with dNTPs to a final concentration of 400 nM, additional MgCl_2_ to a final concentration of 1 mM, pooled primer pairs to a final concentration of 0.25 μM per primer pair, 1.6 U of Phusion Hot Start Flex DNA polymerase (NEB) made up to 10 μl total volume with nuclease-free water and template. The following thermocycling protocol was used: 95° for 10 min then 25 cycles of 95° for 15 sec, 66° for 30 sec then 72° for 1 min before a final extension of 72° for 3 min.

Indexing of the amplified loci was performed in the following reaction mixture: high fidelity buffer was diluted to 1× with dNTPs to a final concentration of 80 nM, either M13 or Fluidigm indexing primers to a final concentration of 0.1 μM along with 0.2 U of the Phusion DNA polymerase. Products of the initial amplification protocol were diluted 10× by adding 1 μl of the reaction product to 9 μl of nuclease-free water and 4 μl of the 10× dilution were added with nuclease-free water to bring the whole reaction mix to 10 μl. The following thermocycling protocol was used: 98° for 2 min then 10 cycles of 98° for 10 sec, 66° for 10 sec, then 72° for 20 sec.

To remove any primer-dimer contaminants or excess primer from each sample, the MagJET NGS cleanup and size selection kit was optimized for use in a 1.2 ml MIDI 96-well plate and Ambion Magnetic Stand-96 (all Thermo Scientific) by halving the manufacturer’s recommended reagent volumes. Solutions were mixed by shaking at 1500 rpm for 15 sec using the Illumina High Speed Microplate Shaker.

Following indexing, size selection and pooling, the libraries were concentrated using the Zymo Research Clean and Concentrator 5. The library was then quality controlled using the Agilent 2100 Bioanalyzer system and quantified using qPCR prior to submission for paired-end 150 bp sequencing in the CRUK-CI genomics core facility on either the MiSeq or HiSeq 4000 platforms.

### Computational estimation of mutant shift and mutant proportion

FASTQ files were demultiplexed using the demuxFQ tool. The PANDAseq tool was used to generate a consensus read from paired forward and reverse reads (Masella *et al.* 2012). The M13 barcoding system sequences an “M13” sequence in line with the amplicon which acts as an “anchor” sequence and allows for more effective consensus sequence generation. To control for this, when the Fluidigm CS1/CS2 adaptor system was used, the M13 sequence was added to the 5′ flank of the forward and reverse sequences *in silico* prior to PANDAseq analysis.

Mutant distributions were predicted by shifting locus-specific reference distributions either leftward or rightward. The distribution of reference and mutant distributions mixed at different proportions was used to predict PPC distributions.

mix(x,Φ,shift)=(1−Φ)ref(x)+(Φ)ref(x−shift)

The predicted distribution that best fit the real crypt data were assessed by least squares allowing inference of the mutant shift and Φ.

### Filtering of crypt microsatellite length distributions

For each mouse included in the study, a reference distribution for each locus was generated by amplifying eight technical replicates of wild-type tail DNA and taking a median distribution. An assumption of the mixture model used to infer Φ is that the reference locus is either homozygous or hemizygous. Thus, prior to estimation of Φ, all reference length distributions for each locus in each mouse were plotted and manually filtered from downstream processing if the reference distribution was observed to be heterozygous. It was also noted that high levels of variability were occasionally observed between technical replicates at single loci within the same sequencing run. The source of this variability is unknown but may be related to stochastic effects related to low primer concentration or poor primer performance during amplification and sequencing. To recognize such variability within each sequencing run at all loci, the sum of least squares (threshold = 0.0035) was used to identify and exclude loci with variable reference replicates (Figure S3, B and C).

### Computating wild-type, partial, and wholly populated crypt thresholds

The model of continuous clonal dynamics requires mutant proportions to be separated into counts of wild-type, partial and wholly population crypts for a given measured mutant proportion Φ. By assuming that the Φ values are drawn from known distribution centered on the true value P(Φ), it is possible to control our classification based on equating type I errors. P_WT_(Φ = 0) is represented by a Gaussian distribution centered on zero, where the shape parameters estimated using the YFP-data (wild type) and P_whole_(Φ = Φ_whole_) is represented by a Gaussian distribution fitted to the YFP+ data (whole). P_partial_(Φ) is taken to be a shifted whole Gaussian distribution centered on a partial ratio P_whole_(Φ = Φ_partial_). These distributions, as well as the type I errors, are shown in Figure S3D.

The threshold between wild type and partials is identified by adjusting Φ_partial_ until the probability of finding a wild-type measurement is equal to the probability of an observed value is less than zero. Similarly, the threshold between partials and wholes is identified by shifting Φ_partial_ until the probability of finding a whole crypt with a value less than Φ_partial_ is equal to the probability that an observed value is greater than the Φ_whole_. The corresponding probabilities are shown in Figure S3D. Using this method, we identified Φ_wt-partial_ = 0.181, Φ_partial-whole_(het) = 0.325, and Φ_partial-whole_(hemi) = 0.627 (*e.g.*, X-linked locus in males) and is calculated by setting P_whole_(Φ = Φ_whole_/2).

### Data availability statement

All datasets and code to generate figures uploaded to GSA Figshare. Strains and plasmids available upon request. Supplemental material available at FigShare: https://doi.org/10.25386/genetics.7811645.

## Results

### Multiplex analysis of microsatellite mutations

Initially, the feasibility of detecting changes in microsatellite length was considered by extrapolating from previously reported frequencies for a transgene containing a [CA]_30_ tract (Kozar *et al.* 2013) (Table S1). From this evaluation, multiple changes in tract length for each mouse could be obtained from analysis of 100 crypts per mouse and between 8 and 20 native loci per crypt. Of note, there are 254 [CA]_30_ microsatellites present in the mm9 reference genome.

**Figure 1:**
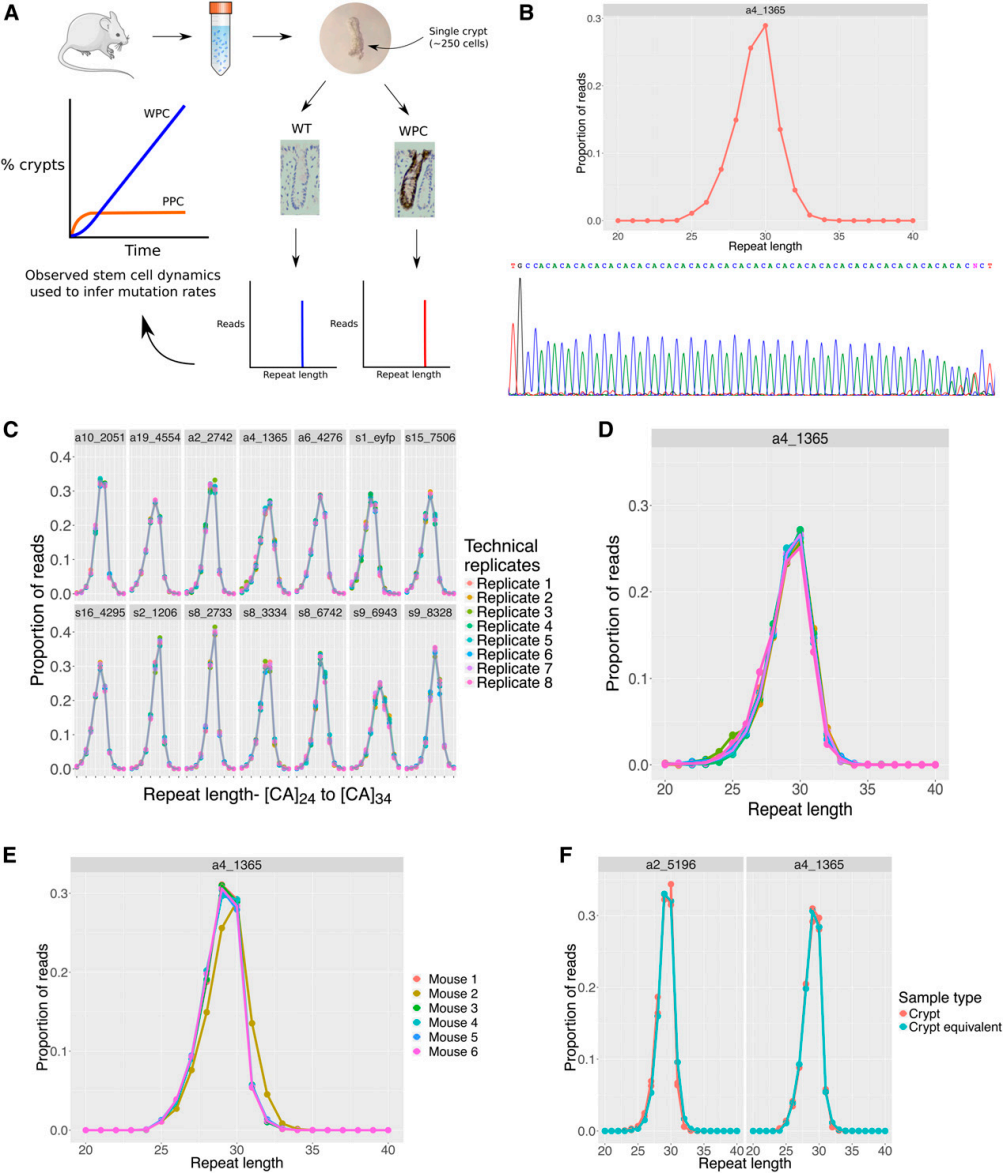
Determining mutant [CA]_n_ microsatellite length using multiplexed Next Generation Sequencing. (A) Schematic of experimental strategy to infer microsatellite mutation rates. Single crypts are isolated from mice of different ages and microsatellite sequencing used to determine their clonal status. The frequency of PPC and rate of WPC accumulation with age is codependent on mutation rate and known stem cell dynamics, namely the functional stem cell number and stem cell replacement rate. (B) Microsatellite locus a4_1365 amplified from bulk DNA extracted from mouse tail and sequenced in singleplex with the validation Sanger sequencing trace shown directly below. (C) Amplification and sequencing of a 14-loci multiplex reaction showing equivalent sequencing fidelity across all loci, including a4_1365. Only 7 of the 14 loci from the multiplex group are presented here. (D) Close up of distribution for a4_1365 shown in (C) demonstrating consistency in read distributions between technical replicates. (E) Biological replicates of the same locus from six different mice amplified in singleplex showing detection of a germline polymorphism in mouse 2. (F) The crypt equivalents used to optimize the protocol produce comparable read distributions to loci amplified directly from crypt lysate; two representative loci are presented from a multiplex group of 14.

We developed a multiplex methodology to accurately detect microsatellite length from pure microsatellite populations. Individual mouse colonic crypts contain ∼250 cells, and, therefore, 500 genomic copies of autosomal loci. This defines the upper limit of the template available. A workflow for the multiplexed amplification of [CA]_30_ microsatellites, prior to sequencing using Illumina technology, was developed using 1 ng of bulk murine DNA; equivalent to the total DNA content of a single crypt, known as “crypt equivalents” (Figure 1A). Primers were designed and run in singleplex PCR and those that produced the expected size band taken forward for Illumina sequencing to confirm that the amplicon contained the correct sequence. Next, the online tool MultiPLX 2.1 was used to determine the optimal combination of primers for use in multiplex groups (Kaplinski and Remm 2015). Multiplexed primers with predicted amplicons in the range of 207–235 bp were confirmed to generate a product that resolved as a single band of expected length using gel electrophoresis before Illumina sequencing to ensure each primer still produced on-target sequence. In addition, the balance in total reads for each primer was assessed in each multiplex group. The multiplex group that produced on-target amplification with the best locus balance was used subsequently (Table S2).

The sequencing data were analyzed on a locus-by-locus basis by measuring the length of sequence between the 5′ and 3′ ends of the microsatellite as specified in the mm9 reference genome (Figure S1A). Accurate microsatellite length estimation required a minimum of 1000 reads per locus (Figure S1, B and C). Conditions were initially optimized using singleplex PCR (Figure S1, D–H and Table S3), which produced accurate, reproducible, and tight read distributions from native [CA]_30_ microsatellites. Using the same reaction conditions, multiplexed amplification of 14 native loci produced consistent read distributions across technical and biological replicates (Figure 1, B–E). Selected loci were validated by Sanger sequencing (Figure 1B). The spread in the read-length distribution is notable and likely results from polymerase error during PCR amplification and sequencing. Germ-line variation between mice was observed in tail DNA at five different loci, confirmed by Sanger sequencing, indicating the ability of the method to detect altered tract lengths (Figure 1E).

To confirm that the use of crypt equivalents for method optimization is a valid approach; the distributions formed from the optimized amplification and sequencing of crypt equivalents were compared with single crypt sequencing showing comparable distributions (Figure 1F).

### Quantifying variant microsatellites in mixtures

**Figure 2:**
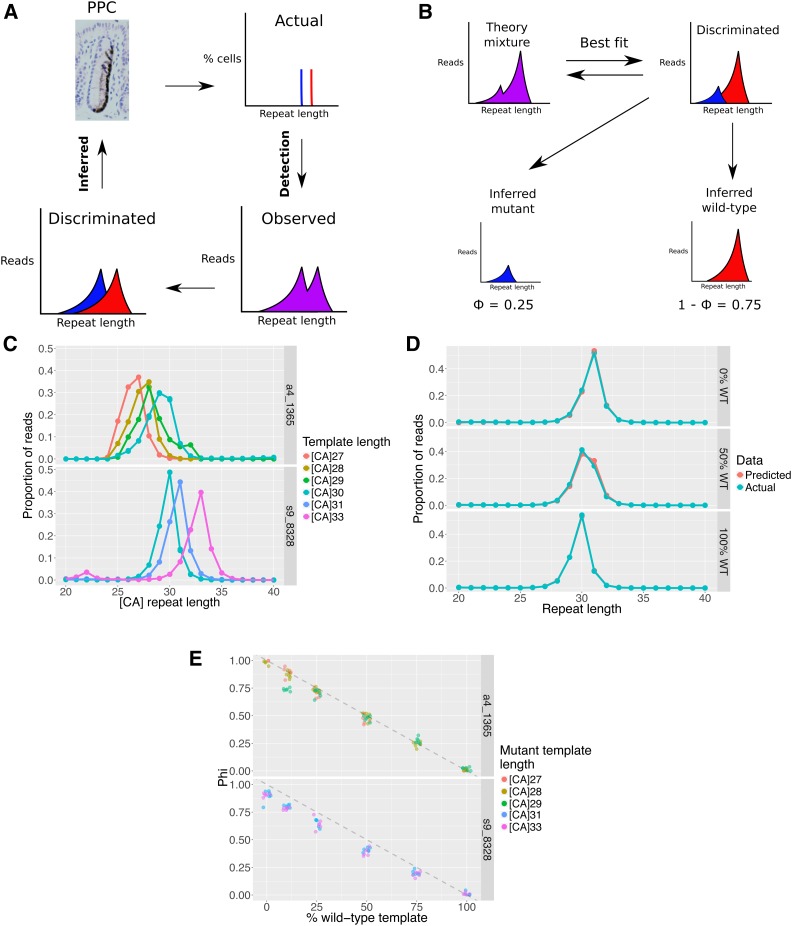
Inferring the proportion of variant microsatellite in mixtures. (A) Detection of transitioning oligoclonal crypts is complicated by read distribution blurring due to PCR and sequencing error. (B) The proportion of WT and mutant microsatellites can be inferred by simulating mixtures in which the mutant proportion (Φ) are simply shifted relative to wild-type proportions (1-Φ) and determining best fit. (C) Microsatellites of differing lengths, in the same DNA context, have broader and less peaked read distributions. (D) The distribution of sequenced s9_8328 plasmid distributions at 0, 50, and 100% WT input (mixed with a [CA]_31_ mutant microsatellite) are compared with the equivalent predicted distributions showing consistency between real and predicted data. (E) Accurate inference of known plasmid mixtures can be attained using NGS at two different loci, with different length mutant microsatellites and at different mixed proportions. Each mutant was mixed with a wild-type [CA]_30_ locus at different proportions with six technical replicates for each mixture. Gray dotted line represents ideal relationship between expected and inferred clonal mixture.

The altered lengths of variant microsatellite that arise somatically are discrete, but must be observed in mixed molecular (for autosomal loci) and cellular populations that also contain the wild-type microsatellite tract. As shown above, due to PCR and sequencing errors associated with microsatellite analyses, the observed tract lengths are not discrete but rather are read as a distribution of lengths. Inevitably, the distributions of variant and wild-type loci will overlap and require discrimination (Figure 2A). Initially, to approximate the read distributions of microsatellite mixtures, a reference distribution for each locus was generated by amplifying eight technical replicates of wild-type tail DNA and taking a median distribution. This reference distribution was shifted to approximate different theoretical mutant distributions. Using the wild-type and theoretical mutant distributions, theoretical “mixtures” of mutant and wild-type distributions at differing proportions could be generated, and least squares analysis applied to determine the best fit (See *Materials and Methods*; Figure 2B). The proportion of variant reads inferred from each mixture was termed Φ.

The applicability of the optimized method for inferring the mutant proportion, depends on the assumption that the shape of the read distributions remains the same for variant tract lengths. To test this assumption, different lengths of synthetic templates corresponding to two native loci were created and cloned into plasmids for propagation in bacteria (Figure S2A and Table S4). These defined templates were used to generate reference distributions documenting the shape of the read distributions with variations in tract length. This demonstrated that as tract length increases the read distribution tends to be broader and less peaked (Figure 2C). However, the effect is incremental such that small changes involving a single [CA] dinucleotide results in a minimal change, indicating that under this condition simple shifting of the reference distribution is a relatively accurate predictor of mutant distributions for a given locus.

To test the ability of the technique to determine different mutant clone size, different lengths of microsatellite were diluted and mixed at different proportions to simulate oligoclonal mutant crypts (Figure S2B). The read distributions obtained following amplification and sequencing of these mixtures are comparable to those predicted from the shift simulations (Figure 2D). Furthermore, the proportion of variant microsatellite in known mixtures was accurately inferred (Figure 2E). This further confirms that any underestimate of the mutant fraction due to altered shapes of peak distribution is insignificant for small changes in microsatellite length.

### Microsatellite length determination in crypts

**Figure 3:**
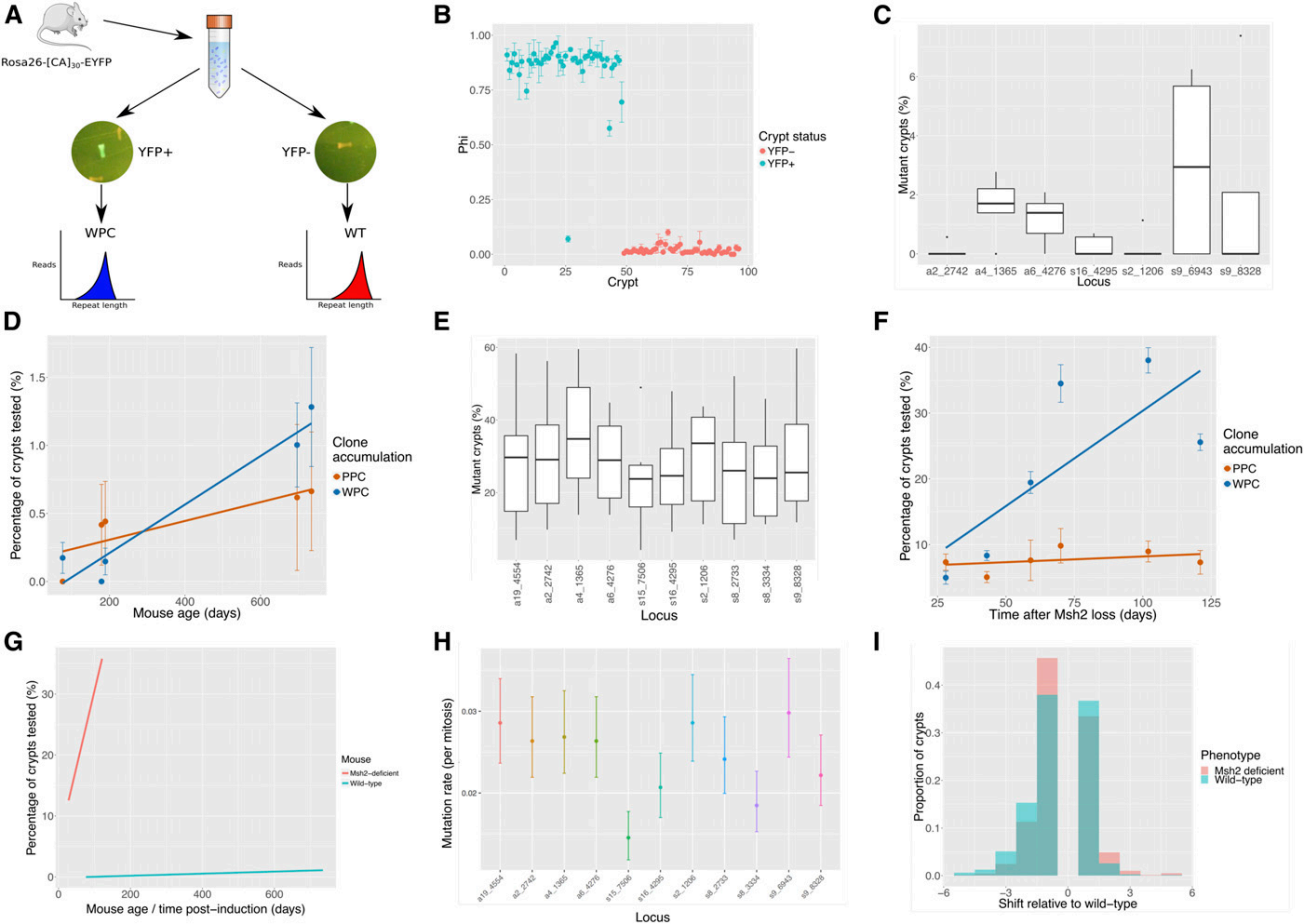
Clonal status and microsatellite mutation rates inferred using microsatellite sequencing of single crypts. (A) Single YFP+ crypts (*i.e.*, mutant WPC) and YFP- crypts (*i.e.*, wild-type crypts) isolated from the intestinal epithelium of the Rosa26-[CA]_30_-YFP mouse provides *a priori* knowledge of microsatellite mutational status. (B) The mutational status of YFP+ and YFP− crypts was correctly ascertained using microsatellite sequencing. (C) Seven loci were analyzed in five wild-type mice ranging in age from 76 to 735 days. The spread of mutant crypts detected at different loci can be observed. (D) Plot showing the percentage of PPCs and WPCs observed in wild-type mice at different ages. The values were calculated by averaging across all loci in each mouse tested (error bars depict the SE of estimates between loci). (E) Eleven loci were analyzed across all six mice at different time points post-induction of *Msh2* gene ablation within the epithelium. The range of mutant crypts observed at each locus across all time points is shown. (F) Scatter plot showing relative changes in PPC and WPC percentages after induction of Msh2-deficiency. The values were calculated by averaging across all loci tested (error bars depict the SE of estimates between loci). (G) The rate of WPC accumulation, dependent on the microsatellite mutation rate, is shown to be significantly higher in the Msh2-deficient crypts when compared with wild-type. (H) Using known stem cell dynamic parameters, the microsatellite mutation rate per mitosis can be calculated across all loci in Msh2-deficient epithelium. (I) Histogram showing the proportion of crypts with different mutant shifts in Msh2-deficient (red) and wild-type (green) crypts.

Previous work has shown that estimates of the frequencies of crypts that are wholly or partly populated by variant clones (WPCs and PPCs, respectively) can be used to describe the clonal dynamics in crypts with knowledge of *de novo* mutation rate (Kozar *et al.* 2013). Reciprocating this relationship, we wished to determine if microsatellite mutation rates read by targeted sequencing can similarly be interpreted using the models that describe crypt clone dynamics. Colonic crypts in which altered microsatellite lengths were known *a priori* were available using the *Rosa26-[CA]_30_-eYFP* mouse, which leads to YFP expression following specific frameshift mutation in a transgenic hemizygous microsatellite (Kozar *et al.* 2013). In this mouse, wild-type [CA]_30_ crypts are YFP^−^ and frameshift mutations in the [CA]_n_ tract that bring the eYFP coding sequence in-frame lead to YFP expression (Figure 3A). Where this in-frame mutation occurs in a stem cell stochastic cell renewal dictates whether the resultant clone populates the entire crypt, recognized as a YFP^+^ crypt, or reverts back to YFP negativity as the clone becomes extinct.

Initial sequencing of the transgenic microsatellite identified high variability in the calculated mutant proportions for YFP^+^ but not YFP^−^ crypts (Figure S3A). This suggests the presence of high amounts of cell free (YFP^−^ wild-type) DNA arising from cell lysis during crypt isolation or contamination by wild-type cells. Multiple phosphate-buffered saline (PBS) washes of each crypt were incorporated into the protocol, and reduced variability for mutant values in YFP^+^ crypts that never reached Φ = 1 (Figure 3B). This suggests that the mutant fraction is consistently underestimated due to the presence of either residual cell free DNA or contaminating wild-type cells (*e.g.*, intraepithelial lymphocytes). Of note, all the YFP^+^ crypts had a +1 dinucleotide shift mutation leading to a [CA]_31_ tract, suggesting that small changes in microsatellite length are more frequent than large-scale changes.

To account for the variation in fractional measurement error in classifying clones, a mathematical model was developed using the mutant distributions observed when sequencing YFP^+^ and YFP^−^ crypts to estimate thresholds that would determine crypt clonal status in native microsatellites (Figure S3D). This allowed for determination of crypt clonal status using a fixed threshold universally applied across all loci and across sequencing runs (see *Materials and Methods*).

### Age-related increases in microsatellite lengths

We next wanted to test if an expected age-related increase in mutation burden could be detected using the above method. Therefore, 744 crypts were isolated from five wild-type mice at different ages (age range 76–735 days, Table S5). Following processing (see *Materials and Methods*), 7 loci from the original 14 that were analyzed across all five mice (five filtered due to length heterozygosity and two filtered due to technical replicate variability), which revealed variation in the mutation burden at each locus. Locus s9_6943 harbored the most mutations, with locus a2_2742 harboring only one mutation across all analyzed crypts (Figure 3C). An age-related increase in WPC microsatellites was observed (Figure 3D) as predicted (Kozar *et al.* 2013). However, an unexpected similar trend was observed for PPCs, which should be present at a constant frequency (Figure 3D). This suggests either that the sum of errors associated with PCR amplification, sequencing, and contaminating wild-type template allows for accurate identification of microsatellites populating all, but not part of, individual crypts, or that there is statistical noise associated with sampling a relatively small number of events.

To further test whether age-related changes in microsatellite mutational burden can be determined by the method, microsatellites were analyzed from the crypts of mice with an increased susceptibility to mutation due to impaired DNA MMR function. The intestinal epithelium engineered to lack Msh2 (a critical component of the MMR pathway) in *Villin^CreER^/Msh2^fl/fl^* mice treated with tamoxifen is predisposed to microsatellite instability, allowing for a higher event rate. At different times following tamoxifen treatment, a total of 720 crypts was isolated from six mice in which epithelial MMR was ablated (Table S6). Out of the 14 loci in the initial multiplex group, 10 were robustly present across all six mice (two loci filtered due to length heterozygosity, and two loci filtered due to technical replicate variability); the rate of mutation observed in each of these loci was relatively uniform when compared with the variation in wild-type mutation rates (Figure 3E). Of the 720 crypts tested, 614 harbored a mutation in at least 1 of the 10 loci tested across all mice. In addition, the frequency of oligoclones remains relatively constant, with an increase in the frequency of monoclonal crypts with time postinduction (Figure 3F) replicating the observations made by Kozar *et al.* (2013). This provides *in vivo* validation that detecting age-related changes in the frequency of microsatellite lengths reflects *in vivo* clonal dynamics, and supports that the wild-type data shown previously is accurate in respect of the accumulation of WPCs but unduly influenced by statistical noise in determining the frequency of the rarer PPCs.

### Inference of microsatellite mutation rates

Next, we estimated the underlying mutation rate of these microsatellites using *a priori* knowledge of stem cell replacement rate and the number of functional stem cells (Kozar *et al.* 2013). Comparing the average rate of clone accumulation in Msh2-deficient and wild-type crypts, demonstrates the degree of increase in age-related mutation burden accompanying loss of Msh2 (Figure 3G). Using the mathematical formula described by Kozar *et al.* (2013), and the derived values of functional stem cell number and stem cell replacement rate, it is possible to infer a microsatellite mutation rate at each locus in both wild-type and Msh2-deficient crypts. An average 388-fold increase in mutation per mitosis rate was observed in Msh2-deficient epithelium compared with wild-type (2.4 × 10^−2^ mutations per mitosis *vs.* 6.2 × 10^−5^ mutations per mitosis). For Msh2 deficiency, the depth of data allows consideration of the microsatellite mutation rate at individual loci, and demonstrates little mutation rate heterogeneity between microsatellite loci in the range 0.0145–0.0298 mutations per mitosis (Figure 3H and Table S7), suggesting only an approximately twofold range in mutation rate between loci.

Of note, the analysis allows inference of the mutant shift in each crypt. In both wild-type and Msh2-deficient crypts, the most likely mutation is +1 dinucleotide or −1 dinucleotide shift within the [CA]_30_, thus producing either [CA]_29_ or [CA]_31_, though other mutant lengths were observed (Figure 3I). This is a consistent observation across all the loci we studied (Figure S3E).

## Discussion

Previously, we have shown that, for any locus, the extent of age-related mutation burden in the colon can be explained by consideration of only three factors, namely the rates of *de novo* mutation, functional stem cell number, and the stem cell replacement (Kozar *et al.* 2013). Here, we develop a method for recognizing age-related mutation burden of native loci, and formally exploit this relationship to infer *de novo* mutation rates. More specifically, we describe a method for the accurate determination of locus-specific microsatellite mutation per mitosis rates by exploiting the clonal architecture of the mouse colon. Our study represents the first estimate of an *in vivo* microsatellite mutation per mitosis rate in wild-type and Msh2-deficient epithelium. Across all loci, there is an increase of two orders of magnitude in [CA]_30_ microsatellite mutation rate in Msh2-deficient epithelium compared to wild type, consistent with previous reports for mismatch repair deficient cell lines (Parsons *et al.* 1993; Yamada *et al.* 2002; Lang *et al.* 2013). This suggests similar susceptibility to [CA]_30_ microsatellite mutation in MMR deficiency at different genomic loci. All the loci studied here are noncoding (3 intronic and 11 intergenic); an expanded multiplexed amplification would enable further study of the spectrum of mutations across different loci within different genomic contexts such as coding *vs.* noncoding (Sammalkorpi *et al.* 2007). This may yet reveal greater diversity in mutation rates in different contexts, and identify candidate sensitive sentinel loci for microsatellite instability. Interestingly, our estimate of an *in vivo* microsatellite mutation rate within wild-type epithelium is approximately half that previously reported in transgenic models (Yamada *et al.* 2002; Kozar *et al.* 2013). This may reflect additional mutagenic processes present at transgenic loci compared with native loci.

Sequencing of single wild-type crypts revealed diversity in the number of mutant events observed at different [CA]_30_ microsatellites. This could suggest mutation rate heterogeneity based on the genomic context of [CA]_30_ microsatellites. However, the relatively modest number of events detected in wild-type mice in the current study prevents comment. A limitation of the technique currently is the need to manually isolate each crypt such that a maximum of ∼300 crypts can be isolated from each mouse. An automated system such as a large particle flow sorter or microfluidic device would allow for substantial scaling of the method.

Our study was limited to loci containing [CA]_30_ microsatellites, which enabled direct comparison of mutation rates and length changes at different loci. Previous work has focused on microsatellite instability in shorter microsatellites with longer, repeating units (Cortes-Ciriano *et al.* 2017). The [CA]_30_ microsatellite mutation rates detected here most likely indicate some of the highest mutation rates occurring within the genome. This makes them particularly amenable to use as sentinel loci for the presence of microsatellite instability in tumor specimens. It may be feasible to identify similar sentinel loci in humans, which could be included in cancer gene sequencing panels either alongside, or replacing, existing repeat sequences used to identify microsatellite instability.

High-depth targeted sequencing enabled accurate quantification of mutant proportion and classification of microsatellite mutation based on length. Plasmid mixing experiments showed the ability of microsatellite sequencing to deconvolute wild type and mutant mixtures as low as 10% variant allele contribution; equivalent to a 20% cellular limit of resolution for autosomal loci. This opens the possibility of the application of this technique outside of epithelial biology to fields such as forensic science. Analysis of the spectrum of microsatellite mutation shifts indicated a clear bias toward single repeat unit loss or gain, with repeat unit loss of two or more being far less likely. This is likely a consequence of the proposed insertion-deletion loop mechanism of microsatellite mutation (Hartenstine *et al.* 2000), and suggests that this mechanism is responsible for the mutations seen in both wild-type and MMR-deficient epithelium, in line with previous reports (Yamada *et al.* 2002).

The method described here utilizes native microsatellites without the need for genetic engineering. This presents a new opportunity for the use of microsatellites as neutral clonal marks that could be used to infer intestinal stem cell dynamics within the human intestinal epithelium. Given their larger size and increased DNA content relative to the murine crypt (approximately eightfold greater), this method should be easily translated to the analysis of human crypts.
